# The impact of abortion denial on women’s empowerment in Nepal (2019–2024): a prospective cohort study

**DOI:** 10.1016/j.lansea.2026.100833

**Published:** 2026-09-03

**Authors:** Nadia G. Diamond-Smith, Diana Greene Foster, Mahesh C. Puri, Rachel Murro, Anupama Ale Magar, M Antonia Biggs

**Affiliations:** a Department of Epidemiology & Biostatistics, University of California, San Francisco, 550 16th St 2nd Floor, San Francisco, CA 94158, USA; b University of California, San Francisco, 1330 Broadway, Suite 1100, Oakland, CA 94612, USA; c Center for Research on Environment Health and Population Activities (CREHPA), Kusunti PO Box 9626, Kathmandu, Nepal

**Keywords:** Empowerment, Decision-making, South Asia, Contraception, Schooling

## Abstract

**Background:**

There has long been an interest in the association between family planning (contraception and abortion) and women’s empowerment, with mixed evidence as to an association and most studies relying on cross sectional data. Within this body of work, less focus has been on how family planning impacts women’s empowerment The aim of this study is to understand the impact of abortion denial resulting in having an unwanted birth on women’s empowerment, measured by education, mobility and decision-making.

**Methods:**

In the Nepal Turnaway Study, we followed women seeking abortions from 2019 to 2020 in Nepal and assessed whether receiving versus being denied an abortion was negatively associated with women’s empowerment over five years (longitudinal cohort). We used linear and logistic mixed effects regression models to assess the effects of obtaining an abortion compared to carrying an unwanted pregnancy to term on education, mobility outside the home and household decision-making power.

**Findings:**

We followed 1742 for 5 years and find that women who did not give birth were more likely to be in school (Risk Difference (RD) = 0.2 starting 18 months post-abortion seeking) and have greater decision-making power over five years (Risk Difference (RD) = −0.2 starting 12 months post-abortion seeking) and both of these continued for the remainder of the time period. We find no evidence that giving birth affects a woman’s freedom to leave her home for shopping or connections to family and friends.

**Interpretation:**

These findings suggest that being able to decide the timing and circumstances of childbearing has wide-ranging effects on women’s empowerment (as measured by schooling and decision-making).

**Funding:**

This study was supported by the National Institute of Health David and Lucile Packard Foundation.

**Research in context**

**Evidence before this study**

The relationship between control over childbearing and women’s empowerment has been theorized but remains underexplored in empirical research. Previous reviews have shown inconsistent evidence linking family planning use to women’s empowerment, with most studies focusing on how empowerment impacts family planning use rather than the reverse. Few studies have rigorously examined how access to abortion services affects empowerment outcomes, particularly in low-resource settings. In South Asia, where women’s mobility and decision-making are often constrained by cultural and social norms, this is especially important. Studies that do exist are often cross-sectional or fail to capture the long-term impacts of abortion access on empowerment. In Nepal, where abortion is broadly legal but often denied, there has been limited investigation into how abortion denial affects women’s life trajectories, leaving critical gaps in understanding the bidirectional relationship between reproductive autonomy and empowerment.

**Added value of this study**

This study leverages longitudinal data from the Nepal Turnaway Study, the first large-scale cohort outside the U.S. to examine the impact of abortion denial on women’s empowerment. By following 1742 women for up to five years, this analysis isolates the effects of being denied an abortion versus giving birth on key empowerment outcomes, including education, household decision-making, and mobility. The findings demonstrate that women who were denied abortions and gave birth experienced significant reductions in educational attainment and household decision-making power compared to women who obtained abortions. There was no evidence that giving birth affected mobility outside the home. These results suggest that enabling women to control the timing and circumstances of childbearing has profound and lasting impacts on their empowerment, particularly in the domains of resources and agency.

**Implications of all the available evidence**

This study provides robust evidence that access to abortion services plays a critical role in advancing women’s empowerment by supporting their educational attainment and decision-making power. The findings challenge assumptions about motherhood automatically enhancing women’s status within the household, suggesting instead that meeting reproductive goals is more protective of empowerment. In contexts like Nepal, where women face significant barriers to reproductive autonomy and low levels of empowerment overall, expanding access to abortion services is essential not only for women’s health and well-being but also for their broader social and economic empowerment. Interventions should prioritize support for women denied abortion services, particularly in maintaining educational opportunities, to mitigate the long-term disempowering effects of carrying unwanted pregnancies to term. These findings contribute to global efforts to improve gender equity and highlight the transformative potential of reproductive autonomy in resource-limited settings.

## Introduction

The relationship between women’s empowerment and use of family planning services, including contraceptive and abortion services, is of much interest, although the direction of the relationship is unclear. A 2017 review found inconsistent evidence as to the association between family planning and empowerment, however, a more recent 2024 review found that women’s agency was positively associated with contraceptive use.^1^^,^^2^ A related review in 2014 explored the literature on empowerment and fertility-related outcomes (number of children, preferences, birth interval and unintended pregnancy), and found overall a positive relationship, with some mixed evidence as well.^3^ A more recent review highlighted the important role of social norms and men’s attitudes in understanding this relationship.^4^ However, in these past reviews, the focus was generally on how women’s empowerment led to family planning use, and mostly included cross-sectional studies. Little research so far has rigorously measured the opposite direction of association, namely, how family planning use impacts women’s empowerment, with a recent review finding limited and mostly low quality evidence, however, an overall trend towards family planning use increasing women’s empowerment.^5^

Empowerment is a complex concept and challenging to measure. Kabeer developed a framework that outlines three domains of empowerment: resources, agency and achievements.^6^ These three dimensions are interrelated and together are part of a process of change that enables women to have power or choices in their life outcomes. Resources, which are conceptualized as pre-conditions, includes material, human and social resources. Agency is the “ability to define one’s goals and act upon them” and includes both observable actions (such as decision-making) as well as a sense of “power within.” Finally, achievements are outcomes related to women or their family’s well-being, for example, health outcomes for themselves or their children.

Empowerment is also challenging to measure because it is a complex, multi-dimensional process which might unfold over long time periods and in myriad ways. Attempts to measure empowerment have often relied on single measures of empowerment that do not cross domains and do not capture the process or multi-dimensional nature. A recent review found that labor force participation and education were commonly measured indicators of “resources”, and household decision-making and bargaining power commonly measured indicators of “agency”.^5^ In South Asia, mobility (women’s ability to leave their home on their own or at all to go to places like a market, health facility, or to see friends) was also a measure of agency used in some previous studies.^7^^,^^8^ Few studies overall measured achievement-related outcomes.^5^

Women who use family planning services are better able to choose if and when to have children.^9^ Spacing or limiting children gives women more opportunities to pursue education, participate in the labor force, and earn an income (resources)^10^^,^^11^(Fig. 1). These opportunities could then, in turn, potentially elevate her status within her household or community, for example, providing her more decision-making power in the household or fewer restrictions on mobility (agency).^12^ This could then increase her achievements, allowing women to advocate or make decisions about seeking heath care.^13^^,^^14^ Obtaining a wanted abortion could also have direct impacts on achievements by allowing women to space their pregnancies and births, improving infant and maternal health, or reducing the economic or emotional burden of having additional children in resource-constrained households (which would have negative impacts on maternal mental and physical health).^15^ On the other hand, in the South Asian context where fertility is highly valued, especially for young, newly married women or in the context of son preference,^16^^,^^17^ it is possible that not having an additional child could reduce women’s agency in her household (if family members wanted a/another child). Women’s empowerment is likely to differ by various socio-demographic and economic factors.^18^ In Nepal, as in many other countries globally, giving birth has significant social, cultural and familial implications and is theorized to be a way of improving women’s standing in their marital household.^19^

**Fig. 1:**
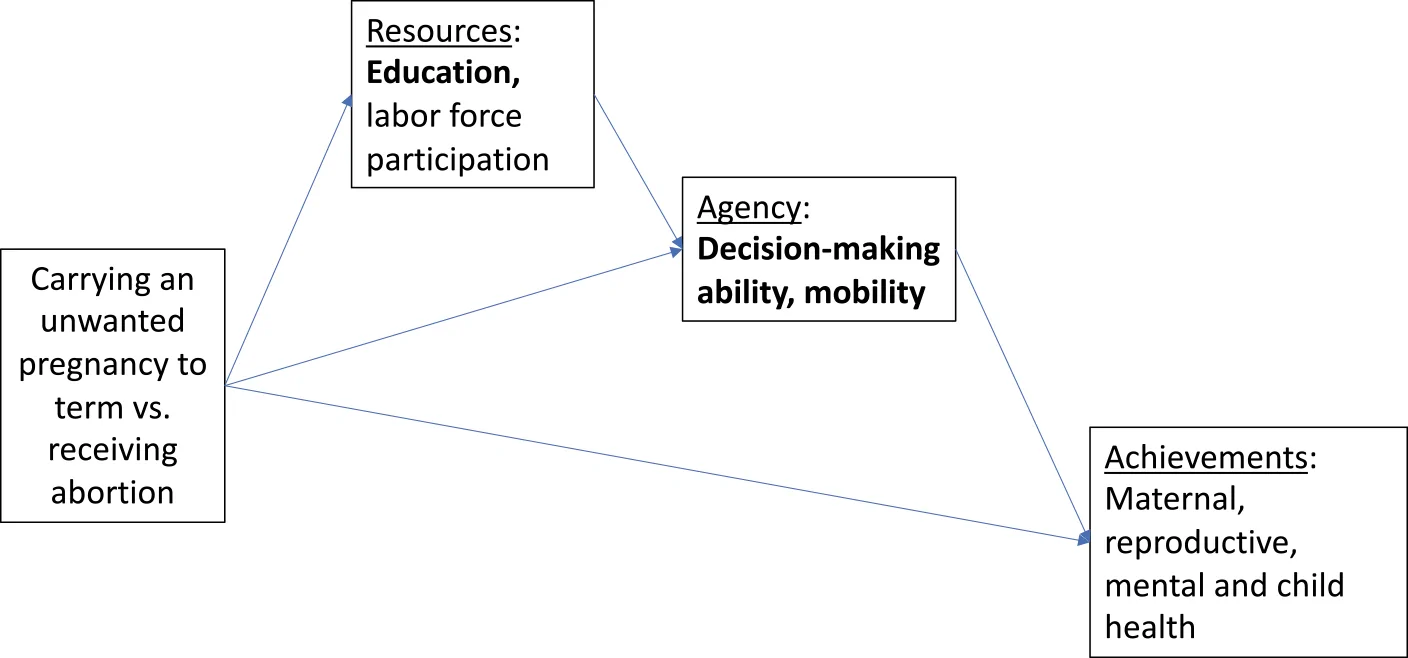
Conceptual model.

Nepal is an interesting case study given relatively recent access to abortion and low levels of women’s empowerment. The Nepal demographic and Health (NDHS) survey in 2022 found that just under half (48%) of women participated in all three domains of household decision-making (either jointly with husbands or on their own).^20^ One of these items was specifically about mobility (freedom to visit a friend or relative). About two thirds (68%) of women said that they were able to make their own decision about this. Generally, men own more household assets than women do and women earn less than their husbands. While girls’ education is catching up to boys’ in Nepal, as it is globally, women’s educational attainment still lags behind men’s. More girls are now in school at the primary level, however, this changes for secondary school, where fewer girls stay in school compared to boys. Women’s empowerment has been shown to be associated with a range of positive health outcomes in Nepal, including in the areas of family planning, child health, intimate partner violence and more.^21^, ^22^, ^23^, ^24^

The data for this analysis come from the Nepal Turnaway Study, a large, longitudinal study across Nepal on the impact of being denied an abortion on various maternal and child health and economic outcomes. In 2018, the parliament passed a separate act known as the Safe Motherhood and Reproductive Health Rights (SMRHR) Act, which guaranteed women’s rights to legal and safe abortion care on wider grounds (up to 28 weeks’ in cases of rape, incest or other special cases).^25^^,^^26^ Despite this, many Nepali women and girls face challenges in exercising their legal right to obtain abortion services.^27^^,^^28^ The goal of this paper is to describe the impact of abortion denial resulting in having an unwanted birth on women’s education, mobility and decision-making over five years using longitudinal data. Given the bidirectionality of the relationship between seeking family planning services and empowerment, this longitudinal study fills a gap in that it can isolate the effect of receipt of family planning services from the effect of seeking services (all women sought care, and only some receive it).

## Methods

We recruited women from 22 clinics and hospitals across Nepal at the time that they were seeking abortion services between 2019 and 2020, forming a longitudinal cohort. Women aged 15 years and older who sought abortion care at participating study facilities and resided in Nepal were eligible for enrollment. All women presenting for abortion care were screened for study eligibility by a point person at the facility. These point people—doctors, nurses, or counselors—completed an eligibility form for every woman seeking an abortion. The form recorded the woman’s age, estimated gestation, provider assessment of eligibility for abortion, and reason for ineligibility, if relevant. If a woman was eligible for the study, the point person at the facility referred her to speak with a trained research staff member who was stationed in a private room at each clinic. The research staff member confirmed study eligibility, obtained written informed consent (for women unable to sign, a thumbprint was obtained), conducted the baseline survey in the clinic using a tablet, and uploaded it to a secure web-based storage platform. In the case of participants under 18 years of age, consent from one parent (either mother or father) and assent from the girl was obtained. Interviewers then conducted in person surveys with participants six weeks later, and at every six months for up to five years. The interviewer conducted the surveys in Nepali, Maithali, Tharu, or Bhojpuri. Each participant received financial compensation of NRs 500 (roughly equivalent to USD $4) for the baseline and each subsequent interview.

All women recruited were seeking an abortion, some received and some were denied the abortion. For the purposes of this paper, we are interested in looking at the impact of having an unwanted birth on women’s life trajectory and thus group all women who received an abortion or ended their pregnancy (whether on the day of recruitment or later) together and compare them to women who gave birth. The vast majority of women who were initially refused abortions but ultimately received one, were able to do so within a few days (delays were due to a doctor not being present that day, or needing someone to accompany them, etc).

We included a continuous time variable to assess whether the birth and abortion group outcomes changed significantly over time. The study group-by-time interaction assessed whether the birth group’s trajectory differed significantly from that of women in the abortion group.

We asked women if they were currently receiving any formal education, with the responses being yes or no. This question was asked every six months for five years.

Two questions measured woman’s involvement in household decision-making. These included who was involved in decisions (1) at home, such as buying goods and (2) about your health care of your family members. Each question included the responses: Both of us, me, my spouse, other, refused, don’t know. These were recoded to include decisions she was involved in (me or both of us) compared to all other responses. These two questions were then summed into a decision-making score that ranged from 0 to 2, with 2 meaning most involved in decisions. These questions were asked at six weeks, 12, 24, 36, 42, 48 and 60 months.

We asked women if in the last year they had been to any of the following places alone: market/bazaar, hospital/clinic/doctor, or outside the village (Yes/No). From this we made a binary variable, where women who reported that they had done none of these were coded as having limited mobility, compared to women who had done at least one of these things. These questions were asked at six weeks, 12, 24, 36, 42, 48 and 60 months.

### Statistical analysis

Given our early findings that women with greater socioeconomic advantages were more likely to receive an abortion,^27^ we employ balancing weights to match covariate distributions across exposure groups at baseline and remove known confounding. Propensity scores were estimated via a multivariable logistic regression model predicting exposure (gave birth versus no birth) based on baseline variables, including: sociodemographic characteristics (age, marital status, caste, education, employment, parity, household income, husband drinking habits), pregnancy characteristics (gestational age, abortion seeking due to fetal anomaly or sex selection, pregnancy intendedness), clinic characteristics (distance from participant home, public/private), health and wellness history (pre-pregnancy health, anxiety/depression, chronic pain, intimate partner violence), and measures of empowerment. Overlap weights, derived from these propensity scores, were applied as probability weights in all analyses. A detailed description of sample weight development and retention/loss to follow up is published in Nepal Turnaway Study Cohort Profile.^29^ There was no difference in loss to follow up by exposure (birth versus no birth).

Counts and percentages were used to describe the data, with chi squared tests used to test for differences between groups. We show both weighted and unweighted descriptives, with exposure balancing weights derived from propensity scores predicting exposure (gave birth). Weighted results apply to artificially balanced sample and thus counts are not shown. We ran multivariable, mixed effects logistic and linear regressions which included random effects for the individual to account for repeated measures and robust standard errors to accommodate clustering by recruitment facility. Models included study group (gave birth versus no birth) as the primary exposure, time in months, and group by time interactions. We tested for the best model fit using a likelihood ratio test to determine if a linear, quadratic or cubic time term best represents the empowerment trajectories over time. We present the marginal predicted probabilities of each outcome by study group to visualize the trends over time and test if the study groups differ significantly at 12-month time points over the course of follow-up.

### Ethical approval

Ethical review and approval was granted by The University of California, San Francisco Human Research Protection Program (18-25863) and the Ethics Committee of the Nepal Health Research Council (704/2018). Informed consent was obtained from all participants prior to study participation.

### Role of the funding source

The funders had no role in the study design, data collection, data analysis, interpretation, or writing of the report.

## Results

Most women (N = 890) received their abortion on the day that they were recruited, another 577 women received their abortion or reported that they were no longer pregnant in the follow-up visits. For the purposes of this paper, we are interested in looking at the impact of having an unwanted birth on women’s life trajectory and thus group all women who received an abortion or ended their pregnancy (whether on the day of recruitment or later) together and compare them to women who gave birth (n = 275, 16%).

Table 1 presents baseline demographic and empowerment characteristics of the 1742 participants overall and by pregnancy outcome. Overall, one-third of women were aged ≤ 24 years (33.2%), 39.4% were Brahmin/Chhetri/Thakuri, and the majority had secondary education (61.6%). Approximately 30% had one living child and 21% had three or more children. About a quarter (26.7%) reported inadequate household income. In the unweighted sample, women who gave birth differed significantly from those who did not across several characteristics. Women who gave birth were younger (40.0% ≤ 24 years versus 32.0%; p = 0.001), more likely to belong to Dalit/other caste groups (24.5% versus 13.1%; p < 0.001), and more likely to report inadequate income (39.0% versus 24.4%; p < 0.001). They also had lower educational attainment, with higher proportions completing only primary education (23.4% versus 13.9%) and fewer completing secondary or higher education (p < 0.001). There were no statistically significant differences in number of living children or sex composition of children. After applying propensity score weights, baseline characteristics were well balanced between groups, and no significant differences remained (all weighted p-values > 0.999).

**Table 1:** Baseline prevalence of empowerment variables and demographic characteristics, overall and by pregnancy outcome.

	Total N = 1742	Unweighted			Weighted		
		No birth N = 1467	Gave birth N = 275	p-value	No birth	Gave birth	p-value
Age, years							
≤24	579 (33.2)	469 (32.0)	110 (40.0)	0.001	(38.6)	(38.6)	>0.999
25–29	504 (28.9)	415 (28.3)	89 (32.4)		(32.6)	(32.6)	
30–34	380 (21.8)	332 (22.6)	48 (17.5)		(17.8)	(17.8)	
35+	279 (16.0)	251 (17.1)	28 (10.2)		(10.9)	(10.9)	
Caste							
Brahmin/Chhetri/Thakuri	683 (39.4)	594 (40.6)	89 (32.6)	<0.001	(34.5)	(34.2)	>0.999
Hill Janajati	415 (23.9)	346 (23.7)	69 (25.3)		(24.9)	(25.1)	
Dalit/other	259 (14.9)	192 (13.1)	67 (24.5)		(21.6)	(21.7)	
Terai Janajati	378 (21.8)	330 (22.6)	48 (17.6)		(19)	(19.1)	
Education							
None/some formal schooling	279 (16.1)	230 (15.7)	49 (17.9)	<0.001	(17.4)	(17.6)	>0.999
Primary	267 (15.4)	203 (13.9)	64 (23.4)		(21.1)	(21.1)	
Secondary	1069 (61.6)	920 (63.0)	149 (54.6)		(56.9)	(56.6)	
Higher	119 (6.9)	108 (7.4)	11 (4.0)		(4.7)	(4.7)	
No. of living children							
0	231 (13.3)	200 (13.6)	31 (11.3)	0.19	(12.1)	(12.1)	>0.999
1	520 (29.9)	438 (29.9)	82 (29.8)		(30)	(30)	
2	621 (35.6)	530 (36.1)	91 (33.1)		(33.8)	(33.8)	
3+	370 (21.2)	299 (20.4)	71 (25.8)		(24.1)	(24.1)	
Sex of living children							
No boys	726 (41.7)	608 (41.4)	118 (42.9)	0.65	(43.9)	(43.9)	>0.999
1+ boys	1016 (58.3)	859 (58.6)	157 (57.1)		(56.1)	(56.1)	
Income adequacy							
Not adequate	463 (26.7)	357 (24.4)	106 (39.0)	<0.001	(34.7)	(35)	0.997
Adequate	1228 (70.9)	1066 (73.0)	162 (59.6)		(63.7)	(63.4)	
More than adequate	42 (2.4)	38 (2.6)	4 (1.5)		(1.5)	(1.5)	
Empowerment variables							
Limited mobility	413 (23.7)	329 (22.4)	84 (30.5)	<0.001	(28.2)	(28.2)	>0.999
Currently in school	188 (12.9)	164 (13.3)	24 (10.8)	0.29	(12.7)	(11.5)	0.658
Household decision-making power				<0.001			>0.999
None	866 (51.4)	707 (49.9)	159 (59.3)		(58.4)	(58.5)	
Some	244 (14.5)	212 (15.0)	32 (11.9)		(12.0)	(12.0)	
More	575 (34.1)	498 (35.1)	77 (28.7)		(29.6)	(29.5)	

*Notes*: Unweighted data are presented as N(column %) for categorical measures and mean(SD) for ordinal measures. Exposure balancing weights derived from propensity scores predicting exposure (gave birth). Weighted results apply to artificially balanced sample and thus counts are not shown.

Focusing in on our main outcome variables, in unweighted models, 13.3% of women were still in school among those that did not give birth and 10.8% among those that did (Table 1). Weighting (using propensity scores) improved this balance; 12.7% of those that did not give birth and 11.5% of those who did were in school. We observed lower decision-making power among those who gave birth (59.3% low, 11.9% medium, and 28.7% high) compared to those who did not give birth (49.9% low, 15.0% medium, and 35.1% high). The difference was statistically significantly different. After weighting, the groups were more balanced and not significantly different (approximately 59%, 12% and 29% across low, medium and high for both groups). Finally, in unweighted models at baseline, 22.4% among those who did not give birth and 30.6% among those that gave birth had limited mobility, and this was statistically significantly different between groups. After weighting, this proportion was the same across groups (28.2%).

There is an overall downward trend in the percent of women who report currently being in school, both overall and by study group, after applying balancing weights. A weighted mixed effects regression analyses with a linear term for time was used to model the schooling outcome. At 18 months after abortion-seeking, women in the “No Birth” group had significantly higher likelihoods of being in school compared to women who gave birth (Risk Difference (RD) = 0.02 starting 18 months post-abortion seeking), see Fig. 2 and Table 2, this difference continued over the 5-year study period at about this magnitude.

**Fig. 2:**
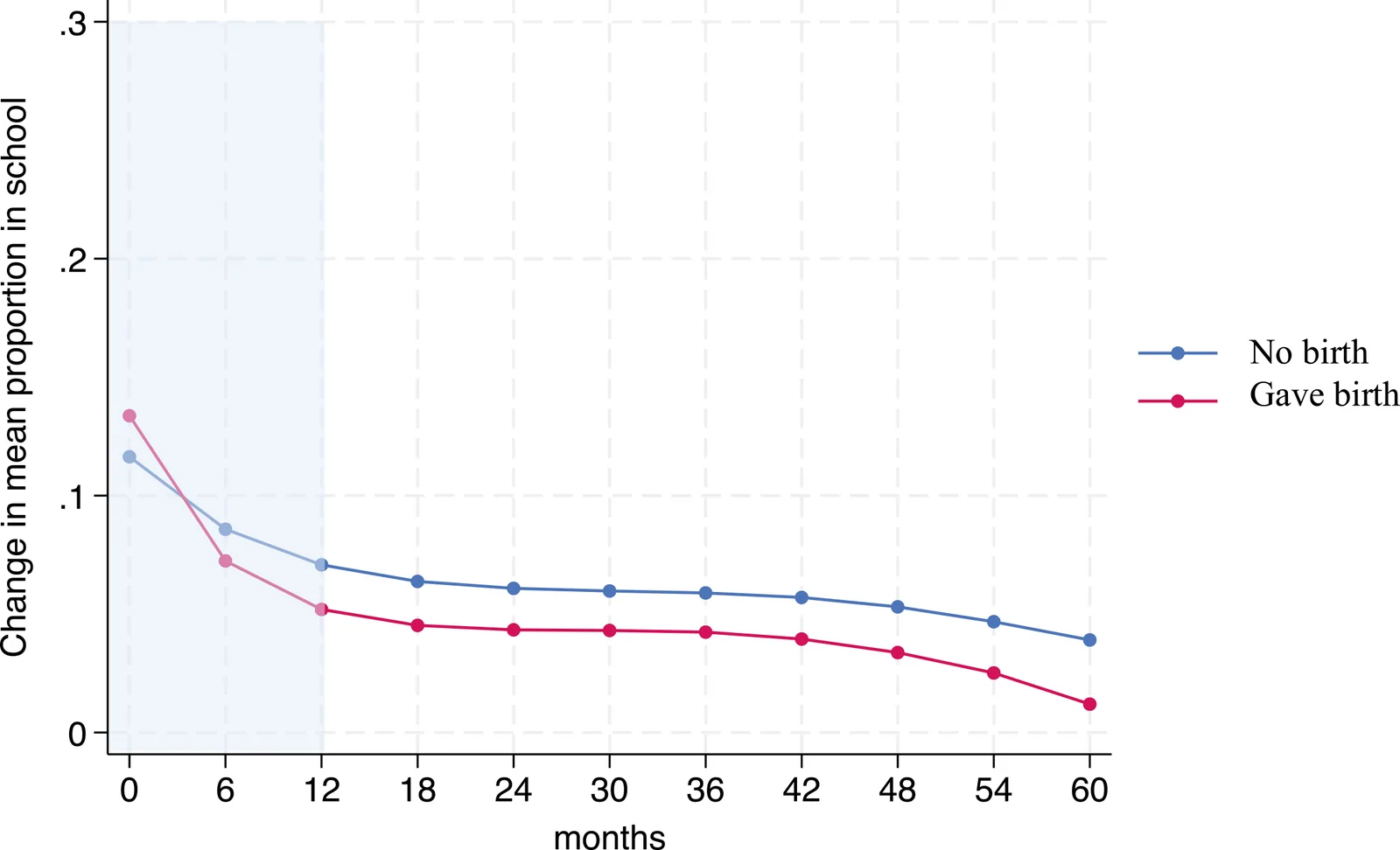
Predictive margins plot of schooling, comparing those who gave birth and those who did not (shaded indicates no significant difference between groups).

**Table 2:** Marginal effect of giving birth on empowerment outcomes, weighted.

	Schooling			Decision-making			Mobility		
	No birth	Gave birth	Risk difference	No birth	Gave birth	Risk difference	No birth	Gave birth	Risk difference
6 weeks	0.12	0.13	0.02 (−0.02 to 0.06)	0.73	0.71	−0.02 (−0.1 to 0.13)	0.29	0.30	0.01 (−0.06 to 0.09)
6 months	0.09	0.07	−0.01 (−0.03 to 0.01)						
1 year	0.07	0.05	−0.02 (−0.04 to 0.00)	1.1	0.85	−0.24∗∗ (−0.39 to 0.1)	0.27	0.29	0.02 (−0.04 to 0.08)
1.5 years	0.06	0.05	−0.02∗ (−0.04 to 0.00)						
2 years	0.06	0.04	−0.02∗ (−0.03 to 0.00)	1.23	0.93	−0.29∗∗ (−0.44 to 0.15)	0.26	0.28	0.03 (−0.02 to 0.07)
2.5 years	0.06	0.04	−0.02∗ (−0.03 to 0.00)						
3 years	0.06	0.04	−0.02∗ (−0.03 to 0.00)	1.24	0.99	−0.25∗∗ (−0.39 to 0.11)	0.24	0.27	0.03 (−0.01 to 0.07)
3.5 years	0.06	0.04	−0.02∗ (−0.03 to 0.00)						
4 years	0.06	0.04	−0.02∗ (−0.03 to 0.00)	1.24	1.05	−0.19∗∗ (−0.32 to −0.05)	0.23	0.27	0.04 (−0.01 to 0.07)
4.5 years	0.05	0.03	−0.02∗ (−0.04 to 0.00)						
5 years	0.04	0.01	−0.03∗ (−0.05 to 0.00)	1.34	1.16	−0.17∗ (−0.33 to 0.02)	0.21	0.26	0.04 (−0.01 to 0.07)

∗∗∗p < 0.000, ∗∗p < 0.01, ∗p < 0.05, using chi 2 tests for difference between groups (gave birth versus no birth) at each time point.

Note: empty cells are because not all variables were collected at all time points.

Women’s involvement in decision-making increased over time for each group, after applying balancing weights. According to our weighted mixed effects regression analyses, with a quadratic time term, over time, women who had no birth had higher decision-making scores starting at 12 months after seeking an abortion, compared to women who gave birth (Fig. 3, Table 2). The risk difference was about −0.02 for the remainder of the study period.

**Fig. 3:**
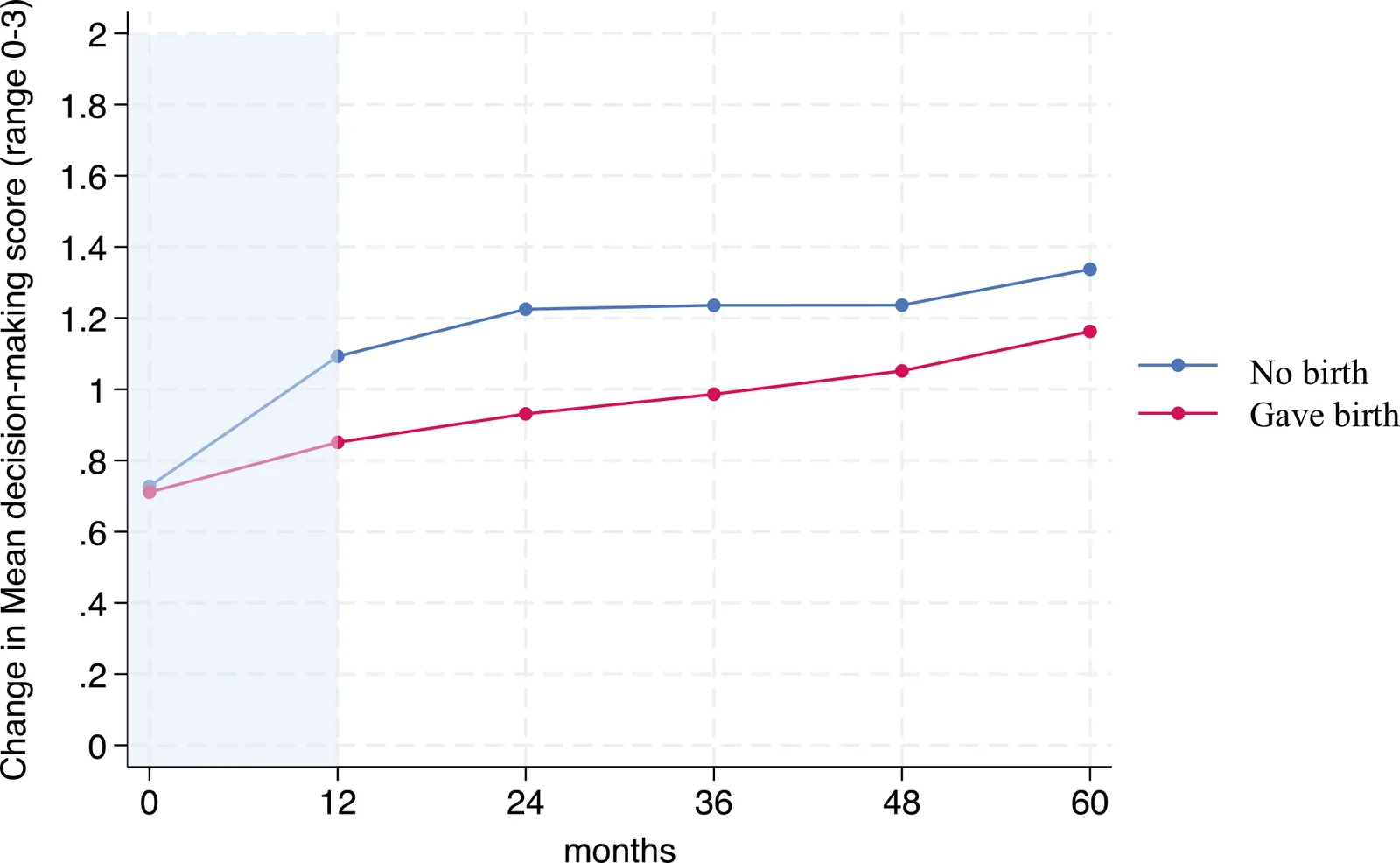
Predictive margins plot of decision-making, comparing those who gave birth and those who did not (shaded indicates no significant difference between groups).

After applying balancing weights, limitations on mobility decreased over time for both groups. While limited mobility was less for those who received an abortion, and we find no statistically significant difference in predicted probability differences from weighted mixed-effects models in mobility between groups at any point during the five-year study period (Fig. 4, Table 2).

**Fig. 4:**
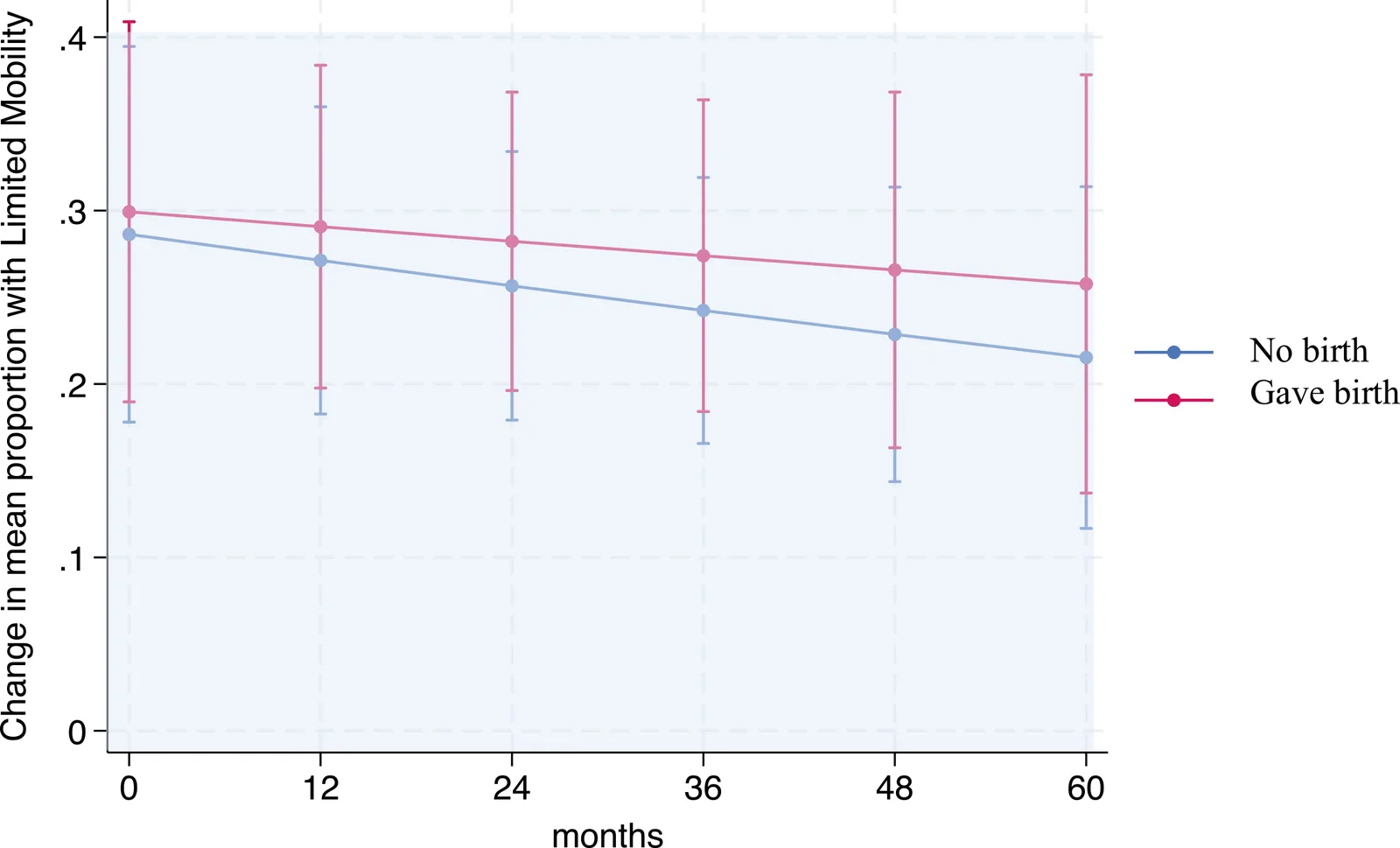
Predictive margins plot of limited mobility, comparing those who gave birth and those who did not (shaded indicates no significant difference between groups).

## Discussion

Being denied a desired abortion reduced women’s school enrollment and her household decision-making five years later. This suggests that having to carry to term an unwanted pregnancy can have lasting impacts on at least two dimensions of women’s empowerment, resources (schooling) and agency (household decision-making). While our study is observational and thus there is the possibility of residual confounding, the longitudinal nature and study design for recruitment, helps lend strength to our findings.

Many pathways could explain these findings. We hypothesize that women who have an additional child at a time that was not ideal for them could have less time to participate in activities such as school or potentially other resource-related activities such as earning wages/working outside the home. Past research in the US found that women who were received an abortion were more likely to be in the labor force and have more economic stability.^30^ Many women in our study stated that they were seeking an abortion because of financial constraints/hardships, so having an additional child could have reduced resources that might otherwise have been spent on school, etc. Additional analysis of this data by Puri et al. indicates that denial of abortion also reduces household financial resources, an outcome which may reduce the resources available to women.^31^

Aside from actual time and monetary impacts, another hypothetical pathway for abortion access to affect women’s empowerment is through the role women must play when they have a child/additional child. There are certain cultural ascribed roles and attributes to motherhood in all settings,^32^ and therefore it might not have been seen to be acceptable for someone with a child, especially an infant, to remain in school. Furthermore, the time required for caring for a child could reduce a woman’s time available to study or attend school. Relatedly, women who are caregiving for small children might be expected to take on certain roles in the household that are less involved in household decision-making. While it has been posited that having a child would increase women’s standing in their households, the evidence about this has been mixed, with previous studies in Nepal showing that women who gave birth had increased exposure to intimate partner violence and did not have consistently increase access to high quality nutritional foods and were not more likely to not eat last in the household (other markers of women’s status).^33^^,^^34^ Murro et al., in another paper in this series using the same data, found that women who gave birth were more likely to experience IPV in the long-term.^35^ Our study adds to these findings to argue that women meeting their reproductive goals may be more important (and protective) for her household decision-making power than giving birth itself. It is possible that reductions in education directly affect decision-making power in the household, both through more educated women being seen to be more able to participate in decision-making by her household members, and through women who have more education having more skills in self-advocacy, communication, negotiation, and other life skills, making them have more agency to demand to participate in household decisions.^36^

About a third of women reported limited mobility, suggested variation in this population and restrictions on mobility still being common. We did not find an effect of abortion denial on mobility. This may be because the practice of women being restricted in their movement outside of the house may be more defined by other household level characteristics related to norms around women’s role and status, and therefore less affected by changes due to childbearing.^37^ There was little change in the proportion of women reporting limited mobility over time, supporting this hypothesis. Mobility being relatively stable over time suggest that either this is a somewhat immutable practice or that our measure might not have captured appropriate domains of mobility. While this is a common measure used to discuss and conceptualize empowerment, there is surprisingly little research on patterns over time or recent research on women’s mobility in South Asia. Some older evidence suggests that mobility might not be a strong indicator of empowerment in this context.^38^^,^^39^

We observe overall trends of decreasing schooling declines over time, increased decision-making, and little change in mobility, as discussed above. We would expect schooling to go down over time as women age and complete their schooling; most women in older age groups would not be in school. The finding that overall decision-making increased over time is in line with past research suggesting that women’s status increases in their marital households as they age.^40^

In this study, we leveraged data from a large, national, longitudinal sample of women over time to understand the impact of abortion access on empowerment. This allows us to see how the trajectories differ between women who gave birth and those who did not, accounting for their baseline differences. However, as with all studies, there are several limitations. First, the two groups of women (those who gave birth and those who did not) did differ at baseline, however, they did not differ significantly by the main empowerment outcomes, except for mobility (which did not end up changing significantly over time). Using propensity score weighing also reduces concerns about this. However, the models used, even when supported by propensity score weighting, cannot account for unmeasured or unknown sources of confounding, which limits causal inference. Another limitation is that not all variables were collected at all timepoints, and some measures had a year of time in between data collection. As mentioned above, empowerment is a complex concept to measure, and, in line with prior scholars on this topic, we pull from various proxy measures that as associated with empowerment, including schooling, mobility and decision-making.^36^^,^^40^^,^^41^ However, other and/or additional measures of empowerment (including income, labor force participation, other measure of agency, etc.) could have been used, which might have led to different interpretations. This study did take place during the COVID-19 pandemic, which could have had implications for our outcomes, most especially formal schooling, which might have been harder to access during COVID-19 restrictions, and mobility, which was limited for other reasons throughout the pandemic. Data was collected from across Nepal, and are likely to be reflective of other South Asian countries with similar norms and practices around women’s empowerment, however, findings might not be generalizable to other countries or places with different gender practices or abortion policies.

This is the first study of its kind outside of the US that aimed to address gaps in evidence about the directionality of effect of control over childbearing and empowerment made possible by the context of Nepal where there are high rates of abortion service denial despite abortion being broadly legal and where women also still have relatively low status in their households and communities. We find that denial can have profound impacts on educational achievement and decision-making abilities of women. Alongside the critical need for expanding access to quality safe abortions for health and reproductive autonomy, interventions are needed that are aimed to provide support to women who are denied abortion care and give birth, so that they can remain in school or continue their education. For example, programs could target women with children to support flexible education programs or childcare support to allow women to go to school. Community level norm change interventions that address women’s decision-making power or mobility could help support women restricted by these gendered norms. While we recognize that the magnitude of the impact found (2%) is relatively small, given the very low status of women in this setting and lack of evidence for successful, cost-effective and scalable interventions to promote women’s empowerment, improvements in access are actionable and feasible ways to help improve the lives of women.

Being able to obtain a wanted abortion has lasting impacts on some domains of women’s empowerment in the near term. These factors will have even longer-term impacts on her empowerment, potentially allowing her to work and earn a better income in the future, negotiate for health services that she and her family need, meet her own aspirations and pass down opportunities and education to the next generation. These findings have important policy implications; by investing in safe and legal access to abortion, and likely other forms of contraception, governments are also investing in women’s economic and social opportunities, with the potential for intergenerational and societal positive effects.

## Contributors

Nadia G Diamond-Smith: **accessed the data, decided to submit the manuscript, analysis, manuscript preparation.**

Diana Greene Foster: study design, funding acquisition, provided feedback on manuscript.

Mahesh C Puri: oversaw implementation of study, provided feedback on manuscript.

Rachel Murro: **accessed the data, analysis**.

Anupama Ale Magar: study implementation, provided feedback on manuscript.

Antonia M Biggs: analysis support, provided feedback on manuscript.

## Data sharing statement

Proposals should be directed to diana.greenefoster@ucsf.edu; to gain access, data requestors will need to sign a data access agreement. Data are available for 5 years at a third party website: https://dataverse.harvard.edu/dataset.xhtml?persistentId=doi:10.7910/DVN/HMOWCA.

## Declaration of interests

The authors report no disclosures of interest.
